# Spent Mushroom Waste as a Media Replacement for Peat Moss in Kai-Lan (*Brassica oleracea* var. Alboglabra) Production

**DOI:** 10.1155/2013/258562

**Published:** 2013-09-09

**Authors:** H. Sendi, M. T. M. Mohamed, M. P. Anwar, H. M. Saud

**Affiliations:** ^1^MARDI Kuching Station, 93050 Kuching, Sarawak, Malaysia; ^2^Department of Crop Science, Universiti Putra Malaysia, 43400 Serdang, Selangor, Malaysia; ^3^Institute of Tropical Agriculture, Universiti Putra Malaysia, 43400 Serdang, Selangor, Malaysia; ^4^Department of Agronomy, Bangladesh Agricultural University, Mymensingh 2202, Bangladesh; ^5^Department of Agriculture Technology, Universiti Putra Malaysia, 43400 Serdang, Selangor, Malaysia

## Abstract

Peat moss (PM) is the most widely used growing substrate for the pot culture. Due to diminishing availability and increasing price of PM, researchers are looking for viable alternatives for peat as a growth media component for potted plants. A pot study was conducted with a view to investigate the possibility of using spent mushroom waste (SMW) for Kai-lan (*Brassica oleracea* var. Alboglabra) production replacing peat moss (PM) in growth media. The treatments evaluated were 100% PM (control), 100% SMW, and mixtures of SMW and PM in different ratios like 1 : 1, 1 : 2, and 2 : 1 (v/v) with/without NPK amendment. The experiment was arranged in a completely randomized design with five replications per treatment. Chemical properties like pH and salinity level (EC) of SMW were within the acceptable range of crop production but, nutrient content, especially nitrogen content was not enough to provide sufficient nutrition to plant for normal growth. Only PM (100%) and SMW and PM mixture in 1 : 1 ratio with NPK amendment performed equally in terms of Kai-lan growth. This study confirms the feasibility of replacing PM by SMW up to a maximum of 50% in the growth media and suggests that NPK supplementation from inorganic sources is to ensure a higher productivity of Kai-lan.

## 1. Introduction

Mushroom cultivation is a lucrative agribusiness. Total mushroom production worldwide has increased more than 6-folds in the last three decades, from about 1.2 million metric tons in 1980 to about 7.3 million metric tons in 2010 [1]. Worldwide, mushrooms are produced on natural materials obtained from agriculture, woodlands, animal husbandry, and manufacturing industries [2]. Some of these wastes include saw dust, banana leaves, peanut hull, corn leaves and husks, sugarcane leaves, rice and wheat straw, cotton wastes, paper wastes, cocoa shells, wheat, bedded horse manure, and various other wastes [3, 4]. The widely used substrate for cultivation of mushroom in Asia is rice straw base, while in the South East Asian countries sawdust is more common nowadays [5]. 

Spent mushroom waste (SMW), otherwise known as spent mushroom substrate (SMS) or spent mushroom compost (SMC), is the leftover after different flushes of mushrooms have been harvested [6]. Normally, at the end of each production cycle, “spent” (used) mushroom substrates are left abandoned or discarded. One of the major environmental problems in the mushroom producing countries remains the treatment and disposal of SMW [7]. About 5 kg of SMW are produced for each kilogram of mushroom [8]. The SMW contains nutrients which could be used for the growth of plants. These materials are generally nontoxic to plants and therefore, could be employed as soil amendment for different crops [6]. The SMW is claimed to be a source of humus formation, and humus provides plant micronutrients, improves soil water holding capacity, soil aeration, and helps maintain soil structure [9]. The SMW is already used in horticulture as a component of potting soil mixes, soil amendment to improve grass in wetlands for remediation of contaminated water, stabilizing severely disturbed soils, bedding for animals, and control plant diseases. Apart from that, SMW can be successfully used as medium for vermiculture, in agriculture or landscape to enrich soils and as a matrix for bioremediation of contaminated soils [2]. If spent mushroom waste is used in proper proportion, it can act as a peat substitute in a soilless culture [10]. As reported by Idowu and Kadiri [11], SMW is an effective soil amendment and conditioner, and addition of SMW has been found to considerably increase yield of many crops. Iwase et al. [12] observed that addition of SMW to soil increased the yield of tomato 7-fold, and the yield of lettuce and radish 2-fold each. A positive impact of using SMW as a growing substrate component on the productivity and quality of different vegetables and other horticultural crops has been confirmed by many researchers worldwide [7, 11, 13–15]. 

Natural soil is acceptable as a growing medium for plants in garden/field, but it is not the right choice for plants in pots or containers because the frequent water demanded by the container plants will cause soil compaction resulting in a tight and brick-like mass [16]. Potting mix is of great importance for quality production of potted plants. Peat is the most accepted and widely used substrate worldwide for potted plant production, and it accounts for a significant portion of growing substrates used for potted plants [17]. Peat has got high water holding capacity, good consistency, low strength, and excellent porosity [14]. However, peat is a natural and nonrenewable resource and its large-scale utilization is of great environmental concern [18]. Moreover, the diminishing reserves of peat have led to price increases which ultimately limit its use [14, 16]. In recent years, researchers are looking for viable alternatives for peat as a component of growth media for potted plants. Several organic waste products such as spent mushroom waste [19], sewage sludge compost [20], municipal solid waste compost [21], animal manure compost [22], and agro-industrial waste compost [23] have shown good results as growth medium instead of peat. All those organic materials sourced from agriculture, forestry, livestock farming, municipal, or industrial wastes are rich sources of different plant nutrients [24]. Moreover, there is a continuous interest in using different organic waste as growing medium and nutrient source for plants due to rising consciousness of environmental issues [25]. 

In Malaysia, mushroom has been grown since early 1930 in Penang [26]. With the increasing production of mushroom, the massive amount of unused spent mushroom waste is of a huge concern. Since information on the use of spent mushroom waste as soil amendment or part of soilless media is still scarce in Malaysia, this study was deemed necessary. This present study was, therefore, designed with an ultimate aim to determine the feasibility of the replacement of peat moss by spent mushroom waste and to evaluate the effects of spent mushroom waste alone and in combination with commercial peat moss on the growth of Kai-lan (*Brassica oleracea* var. Alboglabra), a popular leafy vegetable in Malaysia. Special emphasis was given towards the feasibility of total replacement of peat moss by spent mushroom waste.

## 2. Materials and Methods

### 2.1. Experimental Site

A pot experiment was conducted at Vegetable Unit, University Agriculture Park (TPU), Universiti Putra Malaysia, Malaysia (3°00′21.34′′N, 101°42′ 15.06′′E, 37 m elevation). The local climate was hot, humid, and tropical with plentiful rainfall. Laboratory analysis was done at the Plant Physiology and Postharvest Laboratories, Department of Crop Science, Universiti Putra Malaysia, Malaysia.

### 2.2. Plant Material

Kai-lan (*Brassica oleracea* var. *Alboglabra*), also known as Chinese Kale, was the test crop in this study. The variety used was Kailan 11 which is the most popular and commonly grown variety in Malaysia and matures within 50 days. The seeds were obtained from Green World Seed Company, Malaysia.

### 2.3. Experimental Treatments and Design

Experimental treatments comprised eight different planting media. Spent mushroom waste (SMW) and peat moss (PM) were mixed in different ratios (v/v) and amended with/without NPK fertilizers to prepare different planting media. The treatments included 100% PM (control), 100% SMW, PM + SMW (1 : 1), PM + SMW (1 : 2), PM + SMW (2 : 1), PM + SMW (1 : 1) + NPK, PM + SMW (1 : 2) + NPK, and PM + SMW (2 : 1) + NPK. The experiment was arranged in a completely randomized design (CRD) with five replications. 

### 2.4. Media Preparation and Potting

Peat soil was air dried, crushed, and sieved (<2 mm). Spent mushroom waste was obtained from mushroom production unit, University Agriculture Park. The substrate for producing the oyster mushroom (*Pleurotus sajor-caju*) was rubber sawdust with 10% rice bran, 1% agricultural lime (CaCO_3_), and 0.1% urea. Sieved peat moss was mixed with spent mushroom waste manually until homogeneity before being transferred to plastic pot. The average weight of potting media was set at 1 kg per plastic pot. 

### 2.5. Crop Husbandry

Kai-lan seeds were sown in germination trays containing peat moss as germination media. Two weeks after sowing, seedlings were transferred to plastic pots containing one kg of media prepared as per treatment specifications. Chemical fertilizer NPK Green (15 : 15 : 15) was applied as per treatment at the rate of 1.8 g/pot in 2 splits (0.6 g at 1 week after transplanting and 1.2 g at 3 weeks after transplanting). Watering was done as necessary. Other intercultural operations were performed as per standard practices to maintain the normal growth of plants. No incidence of insect and disease infestations was noticed. Crop was harvested at five weeks after transplanting. 

### 2.6. Data Collection

Data on plant height and leaf number were collected at 1, 3, and 5 weeks after transplanting (WAT). Data on fresh and dry weight of shoot and root, leaf area, root length, and relative leaf chlorophyll content (SPAD value) were recorded only after harvest at 5 WAT. Plant height was measured with a ruler from the surface of the media to the longest leaf tip and was expressed in cm. After harvesting, Kai-lan plants were separated into shoot and root. Number of leaves per plant was counted. Roots were washed in tap water to remove soil particles. Then shoot and root were weighed to measure fresh shoot and root weight. Plant shoot and root were then put into brown envelopes separately and kept in an electric oven at a temperature of 65°C for 72 h. Shoot and root dry weight was measured and expressed in g. Relative leaf chlorophyll content or greenness of leaves was measured in terms of SPAD (Silicon Photon Activated Diode) value using a portable SPAD meter (MINOLTA SPAD-502, Minolta camera Co., Osaka, Japan). Five leaf SPAD readings were taken from the youngest fully expanded leaves and then averaged to give a mean SPAD reading for each replicate. Leaf area was measured by leaf area meter (Licor, Model LI-3100 Area Meter, LI-COR Inc. Lincoln, Nebraska, USA).

Media samples were collected before planting for pH, electrical conductivity (EC), and nutrient analysis. The EC and pH were measured in a 1 : 4 mixture of media and distilled water. The EC was determined using a conductivity meter (CM-20S), and pH using a HORIBA (F-14) pH meter. Total N was measured using a CN analyzer (Sumigraph Model NC-800, Shimadzu Corporation, Japan). The K^+^, Ca^++^, and Mg^++^ were measured using a Polarized Zeeman Atomic Absorption Spectrophotometer (HITACHI 180-60) after extraction in 1 N ammonium acetate (pH 7.0). Phosphate was determined colorimetrically (HITACHI U-1000). 

### 2.7. Statistical Analysis

All data were subjected to analysis of variance using the SAS statistical software package version 9.1 [27]. Significant differences among means were adjudged using Least Significant Difference (LSD) test at *P* ≤ 0.05.

## 3. Results 

### 3.1. Acidity and Salinity of Media

Elemental analysis of different growth media used in this study is shown in Table 1. All the media were slightly acidic in nature with pH values ranging from 5.12 to 6.10. The pH values of different media varied significantly (Table 1). Growth medium containing 100% SMW showed the lowest acidity level (pH 6.10), while 100% PM showed the highest acidity (pH 5.12). When SMW was mixed with PM in different ratios, pH values were higher as compared with 100% PM but lower as compared with 100% SMW. Electrical conductivity (EC) also varied significantly among different media (Table 1). None of the media was saline since the EC values ranged between 0.778 and 1.345 dS/m. The highest and lowest EC values were recorded when SMW and PM were mixed at the ratios of 2 : 1 and 1 : 2, respectively. The EC value of 100% SMW (1.331 dS/m) was much higher than that of 100% PM (0.847 dS/m). Mixture of PMW and PM in equal ratio resulted in intermediate EC values. 

### 3.2. Nutrient Status of Media

All the nutrients, except Mg varied significantly among different growth media (Table 1). Medium having 100% SMW contained the lowest percentage of N (0.34%) compared to other media. On the other hand, N content in 100% PM was the highest (1.02%) among all media. N content decreased gradually with the increasing amount of SMW in the medium (Table 1). In general, P content of all media was low. Among the media, 100% SMW contained the maximum amount of P (0.16%), which was statistically similar to the P content of the medium comprising 2 parts SMW and 1 part PM (0.09% P). The P content of media involving SMW and PM in 1 : 1 or 1 : 2 ratios was similar and very low (0.01% P). P content of 100% PM (0.04%) was only 25% as compared to that of 100% SMW (Table 1). K content of 100% SMW (0.53%) was the highest and that of 100% PM (0.16%) was the lowest among different media. An increasing trend of K content was observed when PM was gradually replaced by SMW in the media. When 2 parts SMW were mixed with 1 part PM, K content was still high (0.42%) and very close to 100% SMW, but when mixed at the ratio of 1 : 2, K content was reduced to 0.30%. Mixture of SMW and PM in equal proportion resulted in an intermediate K content of 0.35% (Table 1). Like N, Ca content also was highest in 100% PM (0.63%) and lowest in 100% SMW (0.51%). When PM was replaced by SMW, K content was reduced as compared with 100% PM. Mixture of SMW and PM irrespective of ratios resulted in statistically similar K content (Table 1). No significant variation with respect to Mg content was noticed among different media (Table 1).

### 3.3. Plant Height of Kai-Lan

Plant height of Kai-lan was recorded at 1, 3, and 5 weeks after transplanting (WAT), and it was significantly influenced by growth media at all sampling dates (Table 2). A positive impact of media amendment with NPK on plant height of Kai-lan was also noticeable at later growth stages. At 1 WAT, Kai-lan grown in 100% PM produced the tallest plant (6.5 cm). Media comprising SMW and PM in the ratios of 1 : 2 without NPK, 1 : 1 amended with NPK, and 1 : 2 amended with NPK also produced statistically similar plant heights with 100% PM. Growth media comprising SMW and PM in equal ratio resulted in the shortest plants (4.7 cm) which were similar to those produced by 100% SMW and SMW mixed with PM in 2 : 1 ratio with/without NPK. It is clear from Table 2 that NPK amendment exerted no influence on plant height of Kai-lan at early growth stage (1 WAT). At 3 WAT, tallest Kai-lan plants were recorded with 100% PM (17.0 cm), and shortest ones with 100% SMW (4.5 cm). Interestingly, all the media containing SMW and PM in different ratios resulted in similar plant height, but a positive and significant impact of media amendment with NPK was observed on Kai-lan plant height at 3 WAT irrespective of mixture ratios. At 5 WAT, plant height varied significantly among growth media and followed a wide range between 5.0 and 22.7 cm (Table 2). Kai-lan grown in 100% PM resulted in the tallest plants (22.7 cm) which was statistically similar to that obtained from Kai-lan grown in media containing SMW and PM in 1 : 2 ratio amended with NPK (20.6 cm). Media containing 100% SMW or both SMW and PM (in different ratios) without NPK amendment produced statistically similar and shortest plants. All growth media, irrespective of SMW and PM ratios, produced taller plants when amended with NPK as compared to treatments without NPK amendment at 5 WAT. 

### 3.4. Leaf Number of Kai-Lan

Number of Kai-lan leaves/plant counted at 1, 3, and 5 WAT was significantly different among growth media (Table 2). At 1 WAT, number of leaves varied within a narrow range between 3 and 4 leaves/plant. Media containing 100% SMW, 100% PM, or their mixture in any ratio amended with NPK produced statistically similar and highest leaves/plant. Mixture of SMW and PM irrespective of ratios and not amended with NPK resulted in the lowest number of leaves/plant (3 leaves/plant). Thus, NPK amendment enhanced leaf production from the early growth stage of Kai-lan. At midgrowth stage (3 WAT), growth medium containing 100% PM performed the best in terms of leaf production (5.7 leaves/plant). Media having 100% SMW or both SMW and PM in different ratios without NPK amendment resulted in the lowest leaf production (<2 leaves/plant). Like early growth stage, NPK treated media produced more leaves/plant as compared with untreated media at 3 WAT. Similar response of leaf number to different growth media was observed in a later growth stage (at 5 WAT). Kai-lan produced the maximum number of leaves (7 leaves/plant) when grown in 100% PM which was statistically similar to those produced (>6 leaves/plant) when grown in media having both SMW and PM in different ratios with NPK treatment. Media containing 100% SMW or SMW and PM mixtures not treated with NPK resulted in the lowest number of leaves (<2 leaves/plant).

### 3.5. Shoot Weight of Kai-Lan

Fresh and dry weight of Kai-lan shoot was recorded during harvest. Data show that both fresh and dry weight of shoot was significantly affected by growth media (Table 3). Plants grown in 100% PM produced the maximum shoot fresh weight (19.16 g/plant) which was statistically similar to that produced when grown in media having SMW and PM in 1 : 2 ratio with NPK amendment (15.86 g/plant). On the contrary, plants grown in 100% SMW resulted in the minimum shoot fresh weight (0.12 g/plant), statistically followed by those obtained from different mixtures of SMW and PM irrespective of ratios without NPK amendment. Media amended with NPK always resulted in higher shoot fresh weight when compared with media without NPK amendment. Shoot dry weight of Kai-lan also followed the similar trend of shoot fresh weight (Table 3). Kai-lan grown in 100% PM resulted in the highest shoot dry weight of 5.66 g/plant. Media containing 100% PM or both SMW and PM in different ratios without NPK amendment resulted in the lowest shoot dry weight of only 0.2–0.3 g/plant. All the NPK amended media produced intermediate shoot dry weight ranging between 2.20 and 3.01 g/plant. From Table 3 it is evident that NPK amendment was advantageous for producing higher above ground biomass of Kai-lan.

### 3.6. Root Weight of Kai-Lan

Fresh and dry weight of Kai-lan root recorded at harvest has been presented in Table 3. Data show that both fresh and dry weight of root differed significantly among growth media. Kai-lan produced the highest root fresh weight (2.90 g/plant) when grown in 100% PM and the lowest one when grown in media containing 100% SMW or SWM and PM mixtures (irrespective of ratios) without NPK amendment (0.36–0.49 g/plant). Media treated with NPK always resulted in higher root fresh weight as compared with media not treated with NPK. Root dry weight also was recorded at the highest (0.29 g/plant) when Kai-lan was grown in 100% PM media statistically followed by media comprising SMW and PM mixed in 1 : 1 or 1 : 2 ratio and amended with NPK. On the other hand, media containing 100% SMW or different combinations of SMW and PM without NPK amendment resulted in the lowest root dry weight of only 0.02 g/plant. Thus, NPK amendment was found beneficial for root growth of Kai-lan.

### 3.7. Root Length of Kai-Lan

Growth medium exerted a significant influence on root length of Kai-lan at harvest (Table 3). Media having 100% PM produced the highest root length of Kai-lan (14.6 cm). Growth media containing 100% SMW or SMW and PM mixtures in 1 : 1 or 2 : 1 ratio without NPK amendment resulted in the lowest root length (ranging from 6.8 to 8.7 cm). Media amended with NPK produced a numerically longer root than media not amended with NPK, but no significant influence of NPK on root length of Kai-lan was evident (Table 3).

### 3.8. Leaf Area of Kai-Lan

A wide and significant variation in leaf area of Kai-lan among different growth media was found (Table 3). The highest leaf area of Kai-lan (303 cm^2^/plant) was recorded with 100% PM, which was statistically similar to that recorded with SMW and PM mixture at 1 : 2 ratio with NPK amendment (250 cm^2^/plant). The lowest leaf area on the other hand, was obtained from the media having 100% SMW and mixture of SMW and PM in different ratios without NPK amendment (2.4–2.8 cm^2^/plant). SMW and PM mixed in 1 : 1 or 2 : 1 ratio with NPK amendment also produced much higher leaf areas (181 and 156 cm^2^/plant) compared to 100% SMW or mixed media without NPK amendment, but still lower than 100% PM. Thus, NPK amendment showed a profound influence on leaf size of Kai-lan. 

### 3.9. SPAD Value of Kai-Lan

The SPAD (Silicon Photon Activated Diode) meter provides a very easy, swift, and nondestructive method for estimating relative leaf chlorophyll content. Thus, higher SPAD value indicates healthier plant. SPAD value of Kai-lan differed significantly among growth media (Table 3). Kai-lan plants grown in medium comprising 1 part SMW and 2 parts PM with NPK amendment showed the highest SPAD value of 50.8; media having SMW and PM in 1 : 1 and 2 : 1 ratios with NPK amendment and 100% PM also resulted in statistically similar SPAD values. Kai-lan grown in 100% SMW resulted in the lowest SPAD value of only 14.8, which was statistically similar with the SPAD values obtained from media having SMW and PM in different ratios without NPK amendment. Media amended with NPK resulted in much higher SPAD values as compared with media not amended with NPK.

## 4. Discussion

Potting medium is an important factor for the production of crop in containers, and component and properties of the potting media are very crucial for higher and quality yields of potted plants. Peat moss has been serving as an excellent substrate for potted plants but, it is a nonrenewable natural resource and its commercial utilization is of huge environmental concern. Moreover, continued use and diminishing reserves of commercial peat have led to price increases which demands consideration of alternative growing materials. In this study, we evaluated spent mushroom waste as a growing substrate to replace peat moss for the production of Kai-lan. 

Chemical properties of growth media are very crucial from the point of view of nutrient availability to the plants. The growth media under study showed that pH range from 5.12 to 6.10 and EC range from 1.345 to 0.778 dSm^−1^ are suitable for normal growth of plant [28]. According to Munita [29], primary nutrients like nitrogen, phosphorus, and potassium as well as secondary elements like Ca and Mg are more available at pH 5.5–6.5 for substrates of organic and mineral origins. As Tariq et al. [16] mentioned, response of plants to NPK is closely associated with pH of potting mixture. Moreover, with the increasing pH, the solubility of many nutrients is reduced and some nutrients are precipitated as solid materials that plant cannot use [30]. In our study, all the media showed EC values in the acceptable range comparative to an ideal substrate. Even the 100% SMW showed EC value of only 1.33 dS/m. But, many researchers reported high salinity of SMW, which is mostly responsible for the limited use of SMW as a potting media [31, 32]. The pH and EC values of 100% PM media were significantly lower than those of 100% SMW media. Mixture of SMW and PM in equal volume resulted in increasing pH and EC values compared to 100% PM, which might be due to a dilution effect. These results are in accordance with those of Eudoxie and Alexender [32], who reported that the combination of commercial PM and SMW resulted in intermediate pH and EC values as compared to PM or SMW only.

Ability to provide essential nutrients to plant is one of the most fundamental criteria while judging the suitability of a growth medium [11, 16]. Among the macronutrients, P and K were recorded in higher amount in SMW compared to PM, but N and Ca concentrations were higher in PM than in SMW. Our findings are in line with Eudoxie and Alexender [32], who noted higher P and K concentrations in SMW than in PM. Eudoxie and Alexender [32] also reported that SMW medium showed greater macronutrient concentration than peat based medium. Medina et al. [7] confirmed that SMW has been shown to increase the nutrient availability of growth media. But the overall nutrient status of SMW was not enough to support normal plant growth without external fertilizer application, which is further confirmed by the poor growth of Kai-lan plants grown in 100% SMW. Although this may not limit the use of SMW as a growing substrate, but will lead to higher cost involvement associated with fertilizer inputs [11]. Indeed, nutrient status of a growing substrate depends on the base material. The SMW used in our study was rubber sawdust based which might influence its nutrient content.

A balanced growing medium that contains an adequate supply of nutrients is essential for plants to attain potential height [16]. Results indicated that using SMW and PM in different proportions as growth media produced different effects on plant height of Kai-lan. It was observed from our study that, compared to other media, SMW alone should be considered low ranking as a potting mix for plant height of Kai-lan. PM alone or SMW and PM mixtures in different proportions with NPK amendment contributed to produce maximum plant height. This might be linked to the positive influence of adequate nutrient availability for plant use. Higher nitrogen content in PM enhanced the vegetative growth of Kai-lan which was ultimately reflected in plant height. These findings are in accordance with those of Eudoxie and Alexender [32] and Awang et al. [33], who also reported differences in plant height among growing substrates. Leaves are important for plants as they are the major site of photosynthesis of green plants where glucose and proteins are manufactured and transpiration occurs [34]. Being a leafy vegetable, leaf number and leaf size are the major yield determinants of Kai-lan. In this study, growth media influenced both leaf number and leaf area of Kai-lan till harvest. Media containing 100% PM or mixture of SMW and PM with NPK amendment performed best in terms of leaf parameter by producing 6 to 7 leaves per plant with a leaf area ranging from 156 to 303 cm^2^/plant. On the other hand, plants were stunted in yellowish conditions and consistently produced two to three small leaves per plant with a leaf area of only 2.5 cm^2^/plant when the plants were grown in 100% SMW or in media without NPK amendment. This might be due to the fact that SMW lacks the essential plant nutrients that plants need to produce and retain leaves. But, media containing 100% PM or both SMW and PM amended with NPK supplied sufficient mineral elements to plants which resulted in a higher number of leaves with more leaf area. Similar findings have been previously reported in okra [11], tomato [32], and *Capsicum spp.* [35].

Growth of Kai-lan in terms of root and shoot weight was much higher when grown in 100% PM and PM and SMW mixtures with NPK amendment compared with that when grown in 100% SMW or PM and SMW mixtures without NPK amendment. Higher nutrient availability to Kai-lan provided by PM and added NPK fertilizers mostly contributed to the better growth of Kai-lan grown in 100% PM or NPK amended media. Low nitrogen content of SMW was the consequence of poor growth of Kai-lan plants grown either in 100% SMW or in media not treated with NPK. Another reason for poor root and shoot growth of Kai-lan grown in 100% SMW may be that the SMW used in this study was rubber sawdust based. Wood products can tie up nitrogen in the soil and cause nitrogen deficiency in plants [36]. Geoffrey [37] also reported that the microorganisms in the soil use nitrogen to break down the wood and instead of the nitrogen going to the plant, it goes to the bacteria. Optimum crop performance is limited by inadequate supply of nutrient elements in growth medium, and fertilizer is the key input contributing to crop productivity by increasing crop yield and improving crop quality [38]. Present findings confirm the superiority of fertilized plants over nonfertilized ones in terms of shoot and root weight. The significant enhancement of crop growth and biomass production with added chemical fertilizer corroborates the report of many others [11, 39]. Root length of Kai-lan was higher when grown in 100% PM as compared with that grown in 100% SMW. This is due to texture of peat moss that is good enough for easy root penetration. Higher nitrogen content of PM might also contributed to longer roots.

SPAD value is proportional to the amount of chlorophyll present in leaf, and a linear relationship exists between SPAD value and leaf nitrogen concentration. Thus, higher SPAD values indicate greener and healthier plants. Foliar SPAD readings of different media were observed once at 5 weeks after transplanting. Only 100% PM and media comprising both SMW and PM amended with NPK showed SPAD readings within sufficiency range (>40) while others showed very poor readings. This might be due to the fact that only 100% PM and NPK amended media supplied sufficient amount of nitrogen to plants which eventually resulted in higher SPAD values. The plants grown in 100% SMW or in media without NPK amendment suffered from nitrogen deficiency resulting in leaf chlorosis and low SPAD values. Altland et al. [40] also confirmed that leaf chlorophyll content is often correlated with the nitrogen content of growth media. Our findings are also supported by a study performed by Altland and Krause [41] in which they observed the differences in SPAD values among growth media.

## 5. Conclusion

In summary, it is feasible to use spent mushroom waste as a growth medium substrate to replace peat moss for pot culture of Kai-lan provided that chemical fertilizers are supplemented to avoid nutrient deficiency. Use of spent mushroom wastes as a substrate component will certainly contribute to their disposal in an environment friendly way and will reduce dependence on peat moss simultaneously. Only, spent mushroom waste alone cannot be used as growing medium because of its low nutrient content; the mixtures composed of 50% spent mushroom waste and 50% peat moss with NPK fertilizer supplementation are the growth media with the most potential due to their physical, chemical, and nutritional features. Thus, it is suggested that spent mushroom waste may be further exploited as a growing substrate and that addition of more than 50% spent mushroom waste to potting mixture is not recommended. For complete replacement of peat moss by spent mushroom waste, an improvement is necessary and can possibly be done by the inclusion of essential nutrient elements in the formulation.

## Figures and Tables

**Table 1:** Chemical composition of study media.

Media	pH	EC (dS/m)	N (%)	P (%)	K (%)	Ca (%)	Mg (%)
100% SMW	6.10a	1.331b	0.34e	0.16a	0.53a	0.51b	0.15a
100% PM	5.12d	0.847d	1.02a	0.04bc	0.16d	0.63a	0.15a
1 : 1 (SMW : PM)	5.92b	0.877c	0.71c	0.01c	0.35bc	0.59a	0.10a
1 : 2 (SMW : PM)	5.89b	0.778e	0.82b	0.01c	0.30c	0.62a	0.13a
2 : 1 (SMW : PM)	5.77c	1.345a	.60d	0.09ab	0.42b	0.58a	0.12a

SMW: spent mushroom waste; PM: peat moss.

Means with the same letter within the same column are not significantly different (*P* < 0.05) according to Least Significant Different (LSD) test.

**Table 2:** Plant height (cm) and number of leaves/plant of Kai-lan as influenced by planting media at different growth stages.

Media	Plant height (cm)			No. of leaves/plant		
	1 WAT	3 WAT	5 WAT	1 WAT	3 WAT	5 WAT
100% SMW	5.0b	4.5c	5.1d	3.8ab	1.7c	1.9c
100% PM	6.5a	17.0a	22.7a	3.9a	5.7a	7.0a
1 : 1 (SMW : PM)	4.7b	5.0c	5.0d	3.3bc	1.8c	1.8c
1 : 2 (SMW : PM)	5.5ab	5.4c	5.8d	3.2bc	1.9c	1.9c
2 : 1 (SMW : PM)	5.0b	5.2c	5.0d	3.0c	1.7c	1.5c
1 : 1 (SMW : PM) + NPK	5.5ab	10.1b	17.5bc	3.9a	4.4b	6.3a
1 : 2 (SMW : PM) + NPK	5.5ab	12.2b	20.6ab	4.0a	4.3b	6.4a
2 : 1 (SMW : PM) + NPK	5.2b	10.7b	16.8c	3.5abc	4.1b	6.1a

SMW: spent mushroom waste; PM: peat moss; NPK: inorganic fertilizer at 18 g/pot; WAT: week after transplanting.

Means with the same letter within the same column are not significantly different (*P* < 0.05) according to Least Significant Different (LSD) test.

**Table 3:** Different growth parameters of Kai-lan as influenced by planting media at harvest (5 weeks after transplanting).

Media	Shoot FW (g/plant)	Root FW (g/plant)	Shoot DW (g/plant)	Root DW (g/plant)	Root length (cm)	Leaf area (cm^2^)	SPAD value
100% SMW	0.11d	0.48d	0.02c	0.02c	6.8e	2.4d	14.8b
100% PM	19.16a	2.90a	5.66a	0.29a	14.6a	303a	42.4a
1 : 1 (SMW : PM)	0.11d	0.49d	0.02c	0.02c	8.7ce	2.8d	17.8b
1 : 2 (SMW : PM)	0.17d	0.37d	0.03c	0.02c	9.8bd	2.4d	19.8b
2 : 1 (SMW : PM)	0.12d	0.36d	0.02c	0.02c	8.2de	2.6d	13.1b
1 : 1 (SMW : PM) + NPK	11.30bc	1.47bc	3.01b	0.16ab	11.3bc	181bc	50.1a
1 : 2 (SMW : PM) + NPK	15.86ab	1.86b	3.39b	0.18ab	11.7b	250ab	50.8a
2 : 1 (SMW : PM) + NPK	8.52c	1.03cd	2.20b	0.08bc	10.1bd	156c	44.7a

SMW: spent mushroom waste; PM: peat moss; NPK: inorganic fertilizer at 18 g/pot; FW: Fresh weight of plant; DW: Dry weight of plant.

Means with the same letter within the same column are not significantly different (*P* < 0.05) according to Least Significant Different (LSD) test.
